# A qualitative exploration of medical students’ placement experiences with telehealth during COVID-19 and recommendations to prepare our future medical workforce

**DOI:** 10.1186/s12909-021-02719-3

**Published:** 2021-08-16

**Authors:** Sabrina W. Pit, Sue Velovski, Krista Cockrell, Jannine Bailey

**Affiliations:** 1 School of Medicine, University Centre for Rural Health, Western Sydney University, 61 Uralba Street, 2480 Lismore, NSW Australia; 2 University Centre for Rural Health, Faculty of Medicine and Health, University of Sydney, 61 Uralba Street, 2480 Lismore, NSW Australia; 3 NSW Rural Doctors Network, 2403 Newcastle, NSW Australia; 4 General Surgery, Northern NSW Local Health District, 2480 Lismore, Australia; 5 Regional Training Hub University Centre for Rural Health University of Sydney, 2480 Lismore, Australia; 6 School of Health Sciences, Western Sydney University, NSW 2795 Bathurst, Australia; 7 School of Medicine, Bathurst Rural Clinical School, Western Sydney University, NSW 2795 Bathurst, Australia

**Keywords:** Workforce, Rural health, Telehealth, Education, Future, Clinical training, COVID-19

## Abstract

**Background:**

Clinical practice is increasingly being digitalised. Little is known about how medical students learn and were exposed to telehealth during COVID-19. This is particularly important if we wish to further improve healthcare access and equity in rural areas and vulnerable populations. This formative study sought to explore the exposure and attitudes of medical students on telehealth and COVID-19 during their rural clinical placement in 2020 and provide recommendations.

**Methods:**

Focus groups were held in August 2020 after completion of a 12-month rural placement. Questions centred around students’ exposure and experiences with telehealth during COVID-19. Data was analysed using thematic analysis.

**Results:**

There has been a clear shift in students now acknowledging the importance of telehealth and, more importantly, expressing a clear wish for telehealth to be embedded in the curriculum starting in their first year. In tandem with this, students expressed the need for their clinical supervisors or hospital teams to have the capability to practice telehealth efficiently as this will improve the telehealth experience and lead to better engagement for both staff and students. Furthermore, it was felt that rural clinicians should play a lead role in telehealth implementation given it is integral to rural practice.

**Conclusions:**

Medical students are more exposed to and more interested to learn about telehealth since COVID-19 and wish to see telehealth training built into their curriculum from the outset of medical school. Themes that emerged from this formative study can potentially assist in planning for telehealth education during and post COVID-19 and inform further telehealth research. Embedding telehealth skills training and guidelines into the medical program, and particularly rural medicine training programs, is essential to prepare the future medical workforce to ensure access and quality patient care during pandemics and also to improve access for rural Australians.

## Background

Healthcare equity during COVID-19 for rural and vulnerable populations has received increased attention [1]. Imposed movement restrictions have had some negative impact on vulnerable and rural populations [2], both patients and healthcare providers alike. Issues include surgery cancellations and a reduction in informal care provisions to loved ones. For example, family members may be unable to accompany elder family members to specialist appointments. Additionally, rural [3], but also urban areas [4], can have extra workforce shortage problems during a pandemic outbreak with subsequent reduced access to and quality of care [3]. The risk of having no staff during a COVID-19 outbreak in rural areas needs to be particularly carefully managed. This is also particularly important if we wish to further improve healthcare access and equity in rural communities [5].

Rural Australia has long recognised the problems with healthcare access and the potential of telehealth as a solution [5, 6]. The International Organisation for Standardisation [7] defines telehealth as: ‘the use of telecommunication techniques for the purpose of providing telemedicine, medical education, and health education over a distance’, while drawing a distinction between this and telemedicine, which is defined as the ‘use of advanced telecommunication technologies to exchange health information and provide health care services across geographic, time, social and cultural barriers’.

Given the long distances of travel for many rural residents to access medical services, rural practices, including specialist practices, have used telehealth consultation many years before governments provided funding for telehealth services due to COVID-19 [6]. For example, the Australian College of Rural and Remote Medicine provided a standards framework in 2016 about telehealth services in remote communities [8]. COVID-19 has led to ‘Forced Innovation’ due to urban and rural people having to change behaviour as patients could no longer physically visit their doctors or other health professionals [9], and health professionals had to find other ways of communicating with their colleagues, which led to increased use of digital solutions. Whilst the problem of not being able to physically visit a healthcare professional has been a common occurrence in rural Australia, many urban Australians and people in other countries faced this problem for the first time with COVID-19. This has led to telehealth now being globally embraced as part of the solution to providing care [1]. Indeed, Fisk and colleagues [6] who compared telehealth implementation in Australia, the UK and the US reported that telehealth is likely to find a stronger place within health service frameworks and lead to increased acceptance among healthcare professionals and patients globally.

‘Forced innovation’ [9] due to the COVID-19 pandemic has also led to a rapid evolution of teaching final year medical students online [10]. As Torda and Perkovic [10] have pointed out we have “*an opportunity to review the curriculum for future doctors, especially its alignment with the skills and capabilities they will need in their careers*.” Telehealth capability is an important feature of future medical training. Iancu and colleagues [11] and others [12] have called for telemedicine curricular activities to be embedded into medical education, and have provided various levels of advice on how medical schools can include medical students in telehealth initiatives. As co-creation is an important feature of e-health implementation [13], it is important to understand medical students’ perspectives on telehealth, who after all form our future medical workforce. Little is known about how medical students learn and were exposed to telehealth during COVID-19 requiring further investigation, although the field is slowly expanding around remote learning. A study from the United States found that first and second year medical students valued the flexibility of remote learning but also acknowledged digital fatigue; they were also less able to actively take part, reducing their capacity to learn clinical skills and their hands-on lab experiences [14]. Additionally, a recent scoping review [15] evaluated the extent and nature of the existing literature on medical student training in eHealth, which included four telehealth papers. The authors identified a paucity of research on competencies that future doctors should develop during medical school in order to practice in an increasingly digital world. Rural clinical schools are ideally placed to teach telehealth for a variety of reasons including rural context due to long distances to health care centres, less access to care and decades of telehealth experience by rural clinicians.

Australian rural clinical schools were set up in the early 2000’s and funded by the Australian government to increase the likelihood of students’ intention to practice in rural areas when they finish [16]. There is increasing evidence that this is the case [16–19], with longer rural placements increasing the likelihood of rural practice [16], but outcomes vary significantly between universities. Rural placements and curricula vary by University and Medical School in terms of types of clinical settings and length. The percentage of students that are allocated to rural placements also varies by University but all focus on giving students the rural experience and understanding of rural healthcare. The government determines rural placements based on rural classifications (RA2-RA5) which is defined by the Australian Statistical Geography Standard (ASGS: RA1-RA5 (Major City, Inner Regional, Outer Regional, Remote, Very Remote) [16]. Currently, 21 universities take part under the Rural Health Multidisciplinary Training Program which funds 19 Rural Clinical Schools with every University providing a unique program. A typical rural setting in Australia can also vary ranging from a rural town with a base hospital and specialist staff to a small remote village with no easy access to care. Clinical placements can vary between Aboriginal Medical Services placements, general practice, community research to routine surgery or oncology rotations in acute care settings.

We have previously reported on final year medical students’ knowledge, attitude and exposure to telehealth when on rural clinical placement prior to COVID-19 [12]. We found that medical student exposure to telehealth varied widely and occurred ad hoc. Students had limited interest in telehealth, although they could see the benefit of using telehealth for things such as scripts, and to reduce social isolation for patients and doctors. Students reported the following as challenges for using telehealth: “legal/liability issues, technology, organisational issues, patient rapport, potential lower quality of care, lack of confidence in clinical ability, and a preference for ‘face-to-face' medicine”[12]. Furthermore, students thought that rural, instead of urban clinicians need to lead the telehealth agenda and that clear telehealth skills training and guidelines were needed. Important, some students perceived that some metropolitan doctors used telehealth to expand their own patient base. We argued pre-COVID-19 that understanding telehealth services business models and actively practicing telehealth skills may help medical students’ in being willing to use telehealth services when they are registered practitioners. This will also increase their likelihood of providing telehealth services in rural or urban areas. In turn, this may also reduce the inequity in rural healthcare access that is currently exacerbated by COVID-19 [12]. Since COVID-19 some studies have been conducted around COVID-19 and medical training such as with basic surgical trainees [20].

COVID-19 has changed this landscape dramatically and the aim of this study is therefore to explore the exposure and attitudes of medical students to telehealth in connection with COVID-19 during their rural clinical placement in 2020; and provide a set of recommendations for embedding telehealth skills training and guidelines into the medical program, and particularly rural medicine training programs.

## Methods

### Study design, participants and recruitment

A formative qualitative study design using focus groups was used. The students were in their final year of a 5-year undergraduate medical degree and completion of a 12-month placement in a rural area. Sixteen students (10 females and 6 males) at Rural Clinical School 1 completed placements in general practice, critical care, paediatrics, medicine, surgery, oncology, Aboriginal health and mental health. Eighteen students (11 female and 7 male) at Rural Clinical School 2 completed similar placements in surgery, general practice, oncology, haematology, Aboriginal health, and mental health. All students completed a rural orientated community research project and were generally young students in their early twenties who had started studying medicine straight after high school.

 All 34 students were invited to take part by email invitation from their student coordinators Participants were known to each other beforehand as they had all spent the last 12 months studying and living in close proximity to each other in a rural area away from their usual urban place of residence. Participation was voluntary. Due to COVID-19 the rate of participation was lower than expected as it was not possible to conduct the focus group face to face and we had access to a limited number of students. Two focus groups were held with 18 (n = 12 and n = 6) final year medical students at two Australian rural clinical schools in August 2020 after completion of a 12-month rural placement. The student perspectives about telehealth were offered from those who experienced telehealth and those who did not as we invited all students who had completed their placements to take part. Students discussed experiences in telehealth in clinics as well as  telephone consultations and video-consultations.

### Data Collection

Open-ended questions were developed based on the literature and in consultation with medical education staff from both rural clinical schools [12]. Students were provided with the definition of telehealth from the International Organisation for Standardisation [7] to ensure a common understanding: *‘use of telecommunication techniques for the purpose of providing telemedicine, medical education, and health education over a distance’, while drawing a distinction between this and telemedicine, which is defined as the ‘use of advanced telecommunication technologies to exchange health information and provide health care services across geographic, time, social and cultural barriers’.*

The following questions were asked:

1. Have you been exposed to telehealth during your medical training or elsewhere?
2. Can you explain if it would be beneficial to learn more about telehealth during your training?
3. How and where do you think this could be built into your training?
4. Do you see a lead role for rural clinicians in telehealth services?
5. Did you have any exposure to other new technologies during your undergraduate training and/or clinical placement (e.g. artificial intelligence, virtual reality, augmented reality)?

The facilitator probed to draw out COVID-19 related issues.

Two focus groups were conducted by KC over Zoom (https://zoom.us/) and ran for 50–60 min each. Zoom was chosen due to COVID-19 prohibiting the researchers conducting face-to-face meetings. The focus groups were transcribed via Zoom and checked for consistency by SWP to ensure accurate transcription.

### Data analyses

An inductive thematic analysis was used [21], using Word to organise the data and identify themes. First, SWP and JB read the transcripts to find differences and similarities which were subsequently discussed. Second, a code book was drafted by SWP and checked by JB independently. Thirdly, SWP read the transcripts again and matched the data with the codebook which was further refined, with input and checking by JB. Initial, themes were identified by SWP and refined by JB. This was repeated until consensus was reached between SWP and JB. The final themes were then presented to SV and KC. Direct quotes were used to demonstrate evidence of the findings. Quotes were selected to represent the data, reflecting patterns and themes. It is unknown whether data saturation was achieved.

### Ethics

Ethics approval was granted by Western Sydney University Human Research Ethics Committee (No: H9989). Prior to the focus group, participants were asked to sign a consent form. All methods were carried out in accordance with relevant guidelines and regulations. Informed consent was obtained from all participants and no participants were under 18.

## Results

Students had varying degrees of experience with telehealth during COVID-19 and to our knowledge limited experience prior to COVID-19 with the exception of a telehealth consultation simulation workshop.

**Fig. 1:**
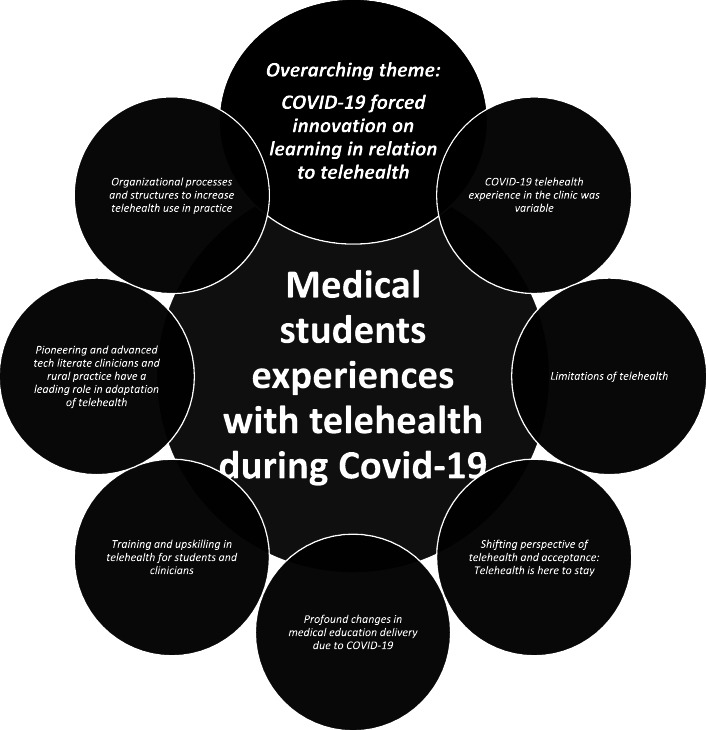
The interconnected themes about rural medical students (n=18) experiences with telehealth during the COVID-19 pandemic

One overarching theme intersected with the other seven themes drawn from the data regarding medical students’ experiences with telehealth in their rural medical training during the COVID-19 pandemic (Fig. 1).

### COVID-19 forced innovation on learning in relation to telehealth

The overarching theme was that COVID-19 has substantially forced immediate changes in the medical education landscape for students in terms of exposure, required knowledge and competencies, training of supervisors, implementation into the real world and the need for integration into practice and medical education.

*Really health is just catching up with technology, like technology is heading down this telehealth pathway … tele anything pathway anyway, and it’s just like COVID is really accelerated the whole process and forced people to actually consider it.*

If COVID-19 had not occurred the students, supervisors and other clinicians’ exposure and experience with telehealth would have been far more limited. Their attitudes towards telehealth were shaped by exposure to telehealth and influenced their views on training needs and organisational processes and structures needed to adopt telehealth. In this sense, COVID-19 forced medical students to consider the use of telehealth and how.

### COVID-19 forced innovation on learning in relation to telehealth

Prior to COVID-19, most students had limited experience with telehealth during their clinical training. During COVID-19, students reported different experiences in the general practice setting versus the hospital. The experience also varied by general practice, with some being exposed to telehealth in general practice whilst others were not. Some of those that did have telehealth experience during COVID-19 reported negative experiences as the GPs were not able to involve them in the consultation process. This was mainly because medical students could only hear one side of the conversation from the GP’s perspective. Students also reported that telehealth consults were shorter, so it was more difficult for their learning when compared to sitting in with face-to-face consultations.

*It’s been used in recently, but I have not seen it.*

*I had my GP rotation during COVID so we had a lot of Telehealth, about a third to a half of them were with the patient over the phone. As a medical student that was a bit hard as it was hard to hear the patient but it was interesting. Different*.

*Sometimes like the patient couldn’t hear him < the GP > well, if he put them on speaker phone so he took them off speakerphone. And I couldn’t hear half the conversation.*

*And the tele health conversation is a bit shorter. So I can’t really follow along as well as what the whole picture was.*

*Pre-COVID I had never had any < telehealth training>, I don’t think, um, but then during COVID…. when I went back and finished my GP time I was with the GPs and some of them did do telehealth.*

*I was always a very passive participant. My GP never asked me to like speak to the patient. They always acknowledged that I was there and gain consent from the patient for me being there because obviously like they couldn’t see me. So it’s important that they know that I’m there because they’re sharing personal things.*

Students felt that Skype handover in the hospital did not work well because a lot of people had trouble with the technology, especially because it was not their own equipment, they were hot-desking, and staff had to use a login every time they wanted to use the computer. This resulted in people not turning up for handover, being less engaged compared to a face-to-face handover, or just skipping over things to save time.

*I think my experience with that and I think their experience of the other medical teams with that is not so good, actually. It’s just that I think a lot of people have troubles with the technology.*

*Time is wasted with someone not muting themselves. Everyone has to repeat themselves all the time. The connection is bad. And I think that can result in suboptimal care, sometimes in complex patients.*

### Limitations of telehealth

Students thought that telehealth was limited for complex patient care especially when technology is not working properly. They also felt it was limited in terms of not being able to do physical examinations or for mental health issues when the patient did not have a quiet place to discuss sensitive issues. Timewasting was also considered as students observed that some patients still needed to come in for a face to face consultation after a teleconsult with a general practitioner. On the flipside, they thought that it was useful for getting test results, ‘get some paperwork sorted’ or chronic care, such as diabetes. For patients, students felt that due to geographical distances telehealth was a benefit and increased service availability for patients.

*Um, it’s just, it’s kind of limited like you can examine the patient, it can be kind of hard to talk about sensitive issues like mental health concerns over Tele health. I think because you don’t have that like human connection as much. Um, but it’s really great for things like giving results. Some chronic care, things like diabetes and things like that.*

*But there are a lot of applications which it’s just not; it doesn’t really work for.*

### Shifting perspective of telehealth and acceptance: Telehealth is here to stay

The current students are now clearly open to the benefit and usefulness of telehealth and acknowledge that COVID-19 has played a role in this. This extended to their learning environment as well.

*I never really thought of it < telehealth > as a I just never really thought of it before, to be honest, but I now think it’s a really, really useful way of doing medicine and also learning.*

*When I kind of assumed that like the use of telehealth is going to be ongoing. I mean, who knows what’ll happen COVID wise, but we’ve sort of realized it does work in some applications.*

*Personally, it’s really exemplified the importance of modifying both learning styles and teaching styles, whether you’re at the receiving end of delivering end of it*.

This cohort also clearly thought that the funding for telehealth should be expanded.

*I think prior to COVID a lot of people had doubts about whether or not telehealth would be feasible as an alternative strategy where people can work from home and conduct these telehealth consults from their own homes. I think what this experience has showed us that it has a lot of potential, we probably should be reinvesting a lot more of our efforts into expanding telehealth*.

### Profound changes in medical education delivery due to COVID-19

All students reported placements and structured social event cancellations and changes, but overall, the students understood and did not feel disadvantaged. Instead, students were satisfied with replacement education that was provided or were given the opportunity to study for their medical exams. Some students were given a private room to study. It was also felt that the rural clinical schools had rapidly adapted to COVID-19. The types of replacement education offered ranged between increased study time, hospital rotations being replaced by tutorials and exam preparations with clinical supervisors.

*Yeah, it’s tough to replace that because telehealth, like it was mostly over the phone and wasn’t even, I’m like face-to-face so they just said it wasn’t worth going into essentially yeah*.

*Yeah, I didn’t complain too much because we did have exams coming up, which were moved forward. So I got a lot of time to study but in terms of clinical education I think I missed out on a lot of like GP hours because of it.*

*I could just start to see everyone like attitudes towards the virus change My next rotation was cancelled because they could not accommodate us anymore. But honestly, from my perspective, I didn’t feel that any of my rural time was drastically impacted by it. Instead I got time to prepare for my exams.*

*I personally didn’t feel disadvantaged by the absence of the surgical orientation, I suppose, even in its place. We had tutorials that were organized by the rural clinical School and I think that was good. And I think it probably was arguably more beneficial than actually going into theatres and standing somewhere in the back and observing from a distance bit. So I would say that’s probably more beneficial in that sense. So yeah that is probably more positive*.

*I was okay with that though, because < name > the person that teaches, mental health here was really great she did that, she went above and beyond to provide extra resources for us and we had like a two week intensive course after exams to prepare for our like mental health OSCEs*.

*I did get some tutorials in place of that, like, and even some like one on one tutorials with one of the radonc < radio-oncology > bosses, which is really cool.*

Cancellations occurred for example in terms of students no longer being allowed to sit in with GP consultations, surgical, oncology, haematology, radiology oncology, Aboriginal Health, paediatric and geriatric mental health rotations. Cancellations occurred because patients stopped coming in and “*there was nothing to see”*, or practices were focussed on “*how it would change the kind of day-to-day operations”* but also some practices felt it was not worthwhile coming in for telehealth consultations whereas other clinical supervisors would still have students attend their clinics.

*I wonder what it would have been like, like, I still actually haven’t seen like a telehealth consult and what they do. Yeah, it would be interesting to see the telehealth consults.*

### Training and upskilling in telehealth for students and clinicians

Students also acknowledged that there appears to be a lack of training whilst the technology is clearly there. Importantly they also noted that tech literate clinicians will improve the telehealth experience for students, demonstrating the urgent need and importance of not only upskilling medical students but also clinical supervisors in telehealth skills

*You know the technology exists, but there’s not necessarily enough training for it, and no one really knows how to navigate it. And that directly relates to how much impact with students this might have in relation to telehealth. So, if our supervisor is quite technologically literate, then our experience will likely be positive. Whereas if they’re not likely to understand how this technology works, then your experiences are also going to be quite negative in that sense.*

One student reported that COVID-19 has led to a new type of clinical question involving technology such as telehealth in that GPs would discuss together to weigh up the risks of not calling a patient in for a face-to-face consult or conduct a teleconsult such as: *“listening to the doctors like decide whether they had to bring someone in to examine them or not, like, that’s an interesting question technical question entirely.*

Students also felt that healthcare staff will not use new technology if it is too hard, suggesting that further training and streamlining of technological processes (see below) and training is required.

*In terms of people having to use new technology or having to change the way that they do things. If it’s too hard. They just won’t do it and they’ll just revert back to whatever the easiest thing that they are used to.*

*It’s just, it’s a little bit of a hassle to migrate some, like central functions in the hospital into telehealth capacity. I think a lot of them < hospital staff > started to give up on that, even if it’s like I think a lot of people like will skip it rather than trying to navigate through these telehealth obstacles.*

With regards to their medical education, students reported that medical educators need to be adequately trained in how to teach online; including how to run meetings or educational sessions and how to keep students engaged throughout.

*On the flip side, as an educator, you have that same struggle of trying to deliver information in a meaningful way while having your students to remain engaged and that’s tricky. And that’s not something that’s inherently you know everyone’s good at, I suppose, and it’s not something that anyone’s really taught how to do.*

Students expressed the need for ‘*some good training’* and recommended several factors that they would like to be taught in relation to telehealth and COVID-19 including training in online communication, how to build rapport over phone/video and applications use. They suggested this be built structurally into their clinical training from the outset as one-off, short sessions are not enough to engender confidence in using the technology: *‘Let’s continue trialling rather than just like just random*.’

*If we could be taught. How to do that over the phone and like strategies for dealing with things that might be a bit difficult over the phone like you know we get taught how to develop rapport maybe being taught strategies, how to develop rapport over the phone or videocall with patients. And then, yeah, like the simple stuff like being taught how to use the applications that we use of course would be good.*

*You probably also need to know IT  < information technology > , teaching and skills on communication over the internet. I think a lot of times, we assume knowledge, but in reality, no one actually knows that.*

*We’ll kind of fumble around and get a couple of lessons that are an hour long on how to use Skype for Business and hope that people then adopt that later on. But they don’t because an hour. So yeah, I agree with you on that one good training. Let’s continue much rather than just like just random.*

When asked about other technologies such as artificial intelligence (AI) and augmented reality (AR), students reported that they need to learn the basics first before learning about more complicated technology.

*I think all those things are pretty cool but you know I think starting the basics is probably a good idea, like AR  < augmented reality >  is awesome. But A its expensive but also B there is no point trying to learn how to do AR now and you can’t start a Skype call for example…*.

Students recommended they need to learn how to communicate with patients in a non-judgemental yet persuasive manner about public health issues, vaccinations, social distancing and mask use, this includes non-medical members of their own family unit. On the flipside, they also suggested that patients need training in how to communicate with their clinician in the online/virtual space.

*So we’re being taught how to talk to patients about things like pandemics in a like non-judgmental way but also persuasively, while still respecting them could be really handy because people will always have different opinions that it’s good to be able to have a productive conversation without offending anyone so yeah.*

*What I experienced with this pandemic was that I had to talk to my family a lot because I was like, there’s a native medical person in the family, and you’re expected to speak with a lot of people coming to you for advice when you’re not fully like I am not an epidemiologist or public health physician Trying to handle these kind of new requests from your family or new request from random people you don’t know but to make sure you don’t give them any misinformation. Just because I’m not fully informed.*

*Yeah, remote monitoring devices. He < GP Supervisor > had like an ECG  < electrocardiogram > . And he had this little gadget that you can use to also take it suppose remotely. I don’t know how successful it is remotely and it requires obviously the patient understand how to use it. So there’s a training element there, but he wasn’t using it with everybody. It was select set of patients that he thought would benefit from it.*

Finally, students also discussed that now they have been taught about personal protective equipment and need to learn how to do nasal-pharyngeal swabbing. Whilst this is not a direct telehealth training need, they are important skills in relation to COVID-19 patient management which includes the combination of telehealth and non-telehealth solutions.

*Which is it’s weird to me that we were never taught it before, but when COVID came, it was like yeah we were immediately were taught how to properly put on our PPE  < personal protective equipment > . Um, I guess, things like swabbing like the nasal pharyngeal swamps, um, we also haven’t really been taught, but they are used of course for COVID*.

### Pioneering and advanced tech literate clinicians and rural practice have a leading role in adaptation

Advanced tech literate GPs were felt to be important and students saw telehealth playing an integral role in rural healthcare to fulfil rural patients’ needs and rural health practitioners being able to fulfill that need. They felt that rural practitioners would benefit a lot more than the city.

*But he himself < GP supervisor>, sorry, he was quite I suppose technologically aware and he was really into software and hardware and how to get things talking in a better, more efficient way and what not, but a lot more complex than what I would understand to be honest.*

*I think it’d be really important for rural practice to have telehealth and active telehealth, just because they are so limited by like geography and the distance between themselves and their patients in the practice and other available services. So yeah, I completely agree. I think that’ll be integral to rural health care.*

Furthermore, rural clinical schools were deemed most suited to teach telehealth, as it is integral to the delivery of rural health care and rural clinicians are considered by students as being the pioneers in this field

*I just think, really, they < rural practitioners > probably benefit from it a lot more so that they should be ideally the pioneers for such a thing, and leading everybody forward.*

*I would say rural health centres are probably, you know, more suited position to deliver such courses because it’s more integral to their model of care and their practices, rather than in the city.*

### Organizational processes and structures to increase telehealth use in practice

Students identified both in general practice and in the hospital the importance of required infrastructure to make telehealth successful and that software and hardware needs to be able *‘to speak to each other’* which can be complex. However, students also reported that infrastructures would not be enough without financial incentives to make it successful. Some students also reported that audio was not always working and therefore that their experience was not that great. It was sometimes hard to hear the patient so the GP would take it off speaker, which would lead to training of lesser quality for the medical student.

*And he actually had this infrastructure set in place before COVID. So for him, adopting this was not that complicated like he was just, you know, just business as usual, but just in a different way.*

*One of the major influencing factors that is often not discussed is the financial incentive that was offered from a governmental perspective to subsidize obviously these consultations and that model of care.*

*Any threat to their model of care that might may be imposed by something like telehealth would have been met with resistance and lack of adoption, but soon as you had sort of government saying, yep we will subsidize it, certain types of consultations, then you know the uptake was massive. And suddenly services were being delivered across GPs and everybody was suddenly capable of, you know, delivering telehealth.*

## Discussion

This study explored how COVID-19 has impacted rural medical students’ educational experiences of telehealth and has identified some preliminary recommendations on how to move forward. Preliminary recommendations of this formative work are summarised in Table 1. Overall, students experienced basic telehealth consultations via the telephone or Skype connections or did not experience telehealth at all due to placements cancellation.

**Table 1:** Recommendations to meet training needs of medical students during COVID-19

Recommendations
***COVID-19 specific***
• Learning how to communicate with patients in a non-judgmental yet persuasive manner about public health measures such as social distancing, mask use and vaccinations
• COVID-19 specific learnings to ensure correct information is passed on to non-medical family members
• Personal Protective Equipment use
• Nasal-pharyngeal swabbing
***Telehealth specific***
• Students reported they would like to learn the following and preferably start in 1st year: o how to talk/consult over the phone o strategies on how to build rapport over the phone or videocall o how to use the applications o communications skills over the internet o principles of telehealth o communication technology
• Start with the basics such as principles of telehealth, communication technology, communication training and then move into integrating different technologies.
• Teach medical educators how to teach online, how to run meetings and keep students engaged
• Integrate telehealth training into the entire medical program as one or two hours on how to use SKYPE is not enough in the hope that people will adopt it later on
• Patients require training to be able to communicate with their clinicians
• Rural clinical schools are most suited to develop and teach these courses given their long history, pioneering and telehealth being integral to rural healthcare
***Advanced technology***
• Need to learn the basics first before learning about more complicated technology such as Artificial intelligence and Augmented Reality.

### Impact of COVID-19 on telehealth

Our previous work pre-COVID-19 identified that students’ overall interest in telehealth appeared to be low and that they had limited exposure to telehealth [12]. The major finding in our study was that there has been a clear shift in students now acknowledging the importance of telehealth and, more importantly, also expressing a clear need for telehealth to be embedded in the curriculum starting in first year. In tandem with this, students expressed the need for their clinical supervisors or hospital teams to have the capability to practice telehealth efficiently as this will improve the telehealth experience and lead to better engagement for both staff and students. This shift in recognising the importance of telehealth may have been influenced by the substantially increased exposure to telehealth due to COVID-19.

Despite COVID-19 leading to cancellations of clinical placement experiences for medical students, the students overall felt satisfied with replacement education that was received and understood the reasons. Contrary to our study [14], a survey among U.S medical students in year 1 and 2 felt that remote learning due to COVID-19 had reduced the quality of education and their ability to participate. These students were not in their final year whereas our cohort was. Similar to our findings, a qualitative study [20] conducted among surgeons in an Irish hospital found that COVID-19 had substantially impacted training exposure through cancelled elective surgery for trainees, especially for more junior trainees. Downsides were that trainees felt that reduced surgical training opportunities hindered their career progression opportunities, effective learning and reduced access to senior mentorship. This was contrary to our study results where students seemed satisfied with what they were offered instead of clinical placements. Bearing in mind though that the students have not experienced their clinical placement at a senior level, and hence are only able to compare to the other teaching they were offered. Furthermore, medical students were preparing for their final year exams and are at the beginning of their career and therefore rather lack an exposure to clinical disciplines which may not interfere as such with their career choices. However, surgical trainees also reported positive findings with online training through their National Training Body and opportunities for independent learning (e.g. improved diagnostic ability), research and non-technical skill development.

### The pioneers

Students thought that rural clinical schools were best suited to teach telehealth because it is integral to rural care and considered rural clinicians the pioneers in this field who should lead everybody forward. Indeed, as Camhi and colleagues [22] point out, telehealth training is critical to improve the care for underserved populations. This was echoed by our medical students in the context of rural and remote locations. They felt that rural clinicians have a lead role to play because telehealth is an integral part of rural health care and have been doing it for some time. This attitude was similar to final year medical students in our previous work conducted pre-COVID-19[12]. Governments could therefore consider providing the resources to further expand the knowledge base that already exists in telehealth training for rural medical schools and clinicians.

### Implementation of telehealth

Some initial recommendations are provided in Table 1. Furthermore, a systematic review of systematic reviews identified key recommendations to ensure efficient e-health implementations. The authors provided key-recommendations that were found to be consistent across various healthcare settings and e-health domains [13], including telehealth. The implementation of telehealth in clinical practice is closely linked to the implementation of telehealth training for students as students learn ‘on-the-job’ and clinicians can learn from students and vice versa. Students’ experiences of telehealth can inform the implementation of telehealth in clinical practice and therefore telehealth education for medical students. The evidence provided by students in this formative research study has provided examples of how telehealth can be improved during training but also in practice. Further to training needs, of particular note is that telehealth requires proper infrastructure and funding. Students observed that both due to the lack of staff familiarity with technology, and/ or the adequacy or otherwise of infrastructure was sometimes lacking (e.g. internet bandwidth/broadband speeds that are required for video-consultations). Notably, tech literate clinical supervisors "increase": the likelihood of a positive educational telehealth experience leading to increased medical student engagement. Therefore, it is recommended to consider engaging tech savvy clinicians and rural clinicians in further development of telehealth training. Furthermore, including the end-user (the medical student) in the design of telehealth education to overcome barriers to telehealth education and increase adaptability and compatibility with existing systems and work practices will further enhance telehealth training and uptake.

Several case studies have been presented on COVID-19 related training implementation. For example, a Dutch hospital proposed a COVID-19 telemonitoring care pathway consisting of video consultations for clinical assessment and monitoring of vital parameters [23]. Home monitoring was conducted by patients, who would receive a COVID-19 Box comprising a thermometer, pulse oximeter, blood pressure monitor, and a safety bag (for return of the devices). A physician or their assistant conducted daily video-consultations. They also provided medical, scientific, organizational, and ethical recommendations to guide the design and implementation of telemonitoring pathways. A similar program can be used to design telehealth training pathways for medical students on rural clinical placement for disease outbreaks such as COVID-19. Students can join similar telehealth care pathways when they are on clinical placement in their latter training years, or simulation can be used in their earlier years through designing telehealth clinical cases and scenarios reflecting real-world problems. We note that a rapid review [24] found that there were no formal studies evaluating basic telehealth educational integration or competencies among health professionals and that the content delivered usually included basic telehealth information. We therefore recommend that, moving forward, formal evaluations are built into telehealth education training, especially now the world has rapidly moved to adoption; we expect to see changes in this area.

### Implications

It is understandable that during the rapid daily changes due to COVID-19, GPs and hospitals did not have sufficient time, resources, know-how or headspace to improve the medical student telehealth experience. There is a clear role to play for rural clinical schools to improve the educational telehealth experience and the telehealth capability for rural medical students and their supervising clinicians, especially with the likelihood of continued COVID-19 outbreaks. As Samareee (2020) from the U.K has pointed out [25]: “*both immediate and long-term measures should be put in place to tackle the serious difficulties faced by medical education and health services over the coming months and beyond.*” We call in line with Shravini and colleagues [14] for an increase in telehealth training for students and medical school staff to increase technological literacy. To this effect, Lum et al. [26] have listed clear learning objectives for telehealth training consultation in knowledge, skills and attitudes mapped against the consultation process consisting of: background to telehealth, pre-consultation, start of the consultation, the consultation, the end of the consultation and post-consultation. Additionally, medical students will need to be taught to understand the standards that apply to inter-operability, security and privacy to improve acceptability and implementation of telehealth.

Studies are evolving quickly using innovative technology to improve telehealth medical education such as an interactive virtual surgical rotation for undergraduate medical education, where medical students were involved in surgical teams while remotely observing surgical procedures with audio-visual technology and could still be engaged in different aspects of care delivery, similar to their usual face-to-face rotations [27]. We also propose that medical schools consider the attendance of medical students via online videoconferencing during clinical consultations, where the supervisor, medical student and the patient are located within their own home environment. Whilst this scenario may be some time away, the possibility should be explored further in preparation for future outbreaks.

In relation to this, although students felt that advanced technology should be taught at a later stage and were not as ready to embrace AI or AR as much as telehealth, we argue the case that it is time for medical education to embed this into medical education as well, or at least start planning for this, as it is expected that this will continue to have a rapid impact on care delivery. Similar to our findings in 2018, when students were less ready to embrace telehealth, well-equipped medical students can embrace these changes faster in time of need as was evidenced by our students advocating that a very tech literate GP made a difference in their learning experience and was interesting.

### Limitations

We were unable to clearly distinguish for all focus group participants’ their experiences of telehealth prior to COVID-19, with the exception of one participant reporting students had received a telehealth consultation simulation workshop prior to COVID-19. Students reported they did not know what they had missed out on in terms of medical education as they don’t know what they don’t know. This may have limited their understanding of the impact of COVID-19 on their telehealth training. Additionally, we had access to a limited number of students due to COVID-19. The study took place among final year medical students that were on a rural clinical placement when COVID-19 started, so the findings may not be generalisable to other settings or year groups. Due to the limited number of students and time restrictions placed on the students, we cannot determine whether data saturation was achieved. Although, rich data was provided and we have been able to form some provisional recommendations. Furthermore, comparison with the existing literature in other groups such as surgical trainees [20] also confirms relatively similar experiences which strengthens our findings. It is also noted that final year students form an important group to call upon in times of crisis such as the Assistant In Medicine program that was implemented in Australia[28] or similar programs in the U.K [29]. Hence, our foundational study has added value due to our ability to gather the views of final year medical students on COVID-19 and telehealth. We do not advocate that medical students are experts in telehealth, rather that their knowledge and experiences can be used to draft recommendations to build a foundation of evidence. Consumer input or end-user input can inform successful implementation. The National Health and Medical research Council state that “*Guidelines can only meet the needs of the population if they are developed with meaningful and authentic engagement with consumers*” [30]. However, clearly more research is needed to build on this foundational evidence and the associated recommendations.

### Zoom

A strength of using Zoom for the focus groups was that students could still choose to take part in the focus groups despite physical distancing measures being in place due to COVID-19. As students knew each other well, it was not apparent that group dynamics hindered people to voice their opinions. A downside was that Zoom cannot transcribe 100 % accurately yet so it was paramount for the researchers to double check the recording with the transcriptions and correct where required. Using Zoom and other video-conferencing tools with automated transcribing features for focus group data collection is likely something that will become increasingly common. It is also expected that the transcribing capability will improve overtime.

## Conclusions

Medical students are more exposed to and more interested to learn about telehealth due to COVID-19. Due to their positive and negative telehealth experiences during the pandemic they are now reporting a need for telehealth training to be integrated into their curriculum at the outset of medical school, alongside training for their clinical supervisors so that they are also comfortable and willing to use telehealth. Lessons learned and themes that emerged from the current study can potentially assist in planning for telehealth implementation during and post COVID-19 and inform further research. Embedding telehealth skills training and guidelines into the medical program is essential to prepare the future medical workforce to ensure access and quality patient care during pandemics and also to improve access for rural Australians

## Data Availability

The datasets are not available from the corresponding author due to the consent being provided for participation in the specific study only.

## Abbreviations

COVID-19: Coronavirus Disease of 2019
GP: General Practitioner
U.K.: United Kingdom
US: CoronavUnited States
ASGS: Australian Statistical Geography Standard
ASGS: Australian Statistical Geography Standard
IT: Information Technology
AR: Augmented Reality
AI: Artificial Intelligence
ECG: Electrocardiogram
PPE: Personal protective equipment
